# Stretch-mediated hypertrophy and strength increases and their impact on dynamic balance performance - a randomized controlled intervention study

**DOI:** 10.1038/s41598-026-43038-1

**Published:** 2026-03-09

**Authors:** Stanislav Dimitri Siegel, Mareike Sproll, Lars H. Lohmann, Niklas Lebelt, Anne Marieke Fasold, Astrid Zech, Konstantin Warneke

**Affiliations:** 1 Institute of Human Movement Science and Exercise Physiology, Friedrich Schiller University Jena, Seidelstraße 20, 07749 Jena, Germany; 2 Institute of Human Movement Science, Sport and Health, University of Graz, Graz, Austria; 3 Institute of Human Movement Science, University of Hamburg, Hamburg, Germany

**Keywords:** Musculoskeletal system, Muscle

## Abstract

Optimizing morphological and functional muscle parameters is of crucial importance in sports and rehabilitation. While resistance training is the most common intervention when improving muscle strength as well as general motor function such as balance, this option is not always applicable, calling for sufficient alternatives. One such opportunity is high-volume, high-intensity stretching. While previous research focused on lower leg muscles, this is the first study exploring effects on the quadriceps. Forty-nine recreationally active participants were allocated into a control group and an intervention group, which underwent twelve sessions of 15-min unilateral supervised hip flexor stretch for both legs over 4–5 weeks. Measurements included muscle thickness, isometric leg extension strength, and dynamic balance. Regional stretch-mediated ultrasound muscle thickness increased significantly in the rectus femoris (proximal: η_p_²=0.24–0.41, *p* = 0.002-<0.001; distal: η_p_²=0.14–0.40, *p* = 0.001-<0.001), but showed inconsistent effects in the vastus lateralis (η_p_²=0.18, *p* = 0.008 only in the left leg). Isometric strength effects were significant in 70° (shortened position, right leg: η_p_²=0.09, *p* = 0.04; left leg: η_p_²=0.21, *p* = 0.001), but not at 110° (lengthened position) knee angle. Balance performance improved significantly leg-dependent in anterior (η_p_²=0.13, *p* = 0.02, left leg), posterolateral (η_p_²=0.14, *p* = 0.009, left leg) and posteromedial directions (η_p_²=0.13, *p* = 0.02, right leg). This study provides first results on stretch-mediated muscle thickness and strength increases in the quadriceps with the potential for implementation in balance training, if participation in an active, supervised training program is not feasible. Future research should include additional balance outcomes, other muscle groups and clinical settings.

German Clinical Trials Register ID: DRKS00036510 (03.02.2026).

URL: https://drks.de/search/en/trial/DRKS00036510/details.

**Supplementary Information:**

The online version contains supplementary material available at 10.1038/s41598-026-43038-1.

## Introduction

The development of muscle strength and structural adaptations is fundamental for improving physical performance^1–3^. This is critical not only in fitness and athletic contexts^4–6^, but also in rehabilitation^7,8^ and health-prevention settings^9,10^. Especially in orthopedic rehabilitation (e.g., after a sports injury or femur fractures in the elderly) restoring muscle function (e.g., strength) and structure (e.g., muscle size) is crucial for secondary prevention, thus, minimizing the risk of re-injury^11–13^.

Structured resistance training (RT) is one of the most effective exercise routines to counteract muscle atrophy and improve clinically relevant parameters^14^. Beyond its well-documented effects on strength and hypertrophy^15,16^, RT also plays a crucial role in improving fall prevention programs^17–20^. Positive correlations between lower body strength and balance performance indicated that increased muscle strength could potentially serve as a moderator for improving balance^21–23^. Accordingly, the 2022 “World Guidelines for Falls Prevention and Management for Older Adults” emphasized muscle strengthening activities as a key component of preventive and rehabilitative routines^24^. Although effective in increasing muscle size and function, substantial portions of the general population do not adhere to common RT recommendations, calling for alternative approaches^25^. This seems especially relevant for immobilized populations or patient groups suffering from serious administrative burdens as professional therapy and training facilities are often fully booked^26,27^. Consequently, researchers advocate for accessible and effective training alternatives that can be easily integrated into daily life^28,29^. One such opportunity was highlighted by recent research suggesting high-volume stretching of up to one hour per day as a potential muscle strengthening activity, which provided a potent muscle hypertrophy stimulus without accessibility restrictions^30–33^. While Schoenfeld et al.^34^ stated that large stretching volumes targeting only small muscles (such as the calf muscles) were required to induce meaningful effects (meaning a full-body stretching routine aiming for appreciable increases in muscle strength and size would entail a disproportionate time investment), a valid opportunity for its application stems from the possibility to integrate stretching as a passive intervention into daily routines such as watching TV or working in the office^35^. Accordingly, especially in rehabilitative settings where exercise supervision of active interventions is required, this passive substitution might be of high potential, for instance in fall prevention. However, the potential transferability of stretch-induced adaptations to balance ability—and their relevance for fall prevention programs in healthy and conditioned populations (e.g., in rehabilitative settings following immobilization-induced atrophy) remains speculative^36^. While low volume stretching of just a few minutes was insufficient to cause meaningful adaptations^37,38^, stretching sessions lasting > 15 min and performed at least four times per week produce practically meaningful hypertrophy and strength adaptations^28–40^. Therefore, the lack of significant quadriceps muscle hypertrophy observed in the study by Wohlann et al.^37^ may reflect an insufficient stretching dosage. Beyond volume, Panidi et al.^41^ and Apostopoulos et al.^42^ emphasized the importance of high stretching intensities for sufficient muscular adaptations, meaning that stretching was performed at maximal tolerable stretching pain, at the point of discomfort or maximal tolerable after the onset of pain^41^.

Since no previous study has investigated the effects of appropriate stretching durations (15 min per session, ≥3x per week^40^) on the quadriceps femoris muscle, this study will be the first to address both aspects. Accordingly, we hypothesized that (1) 15 min of stretching per session targeting the knee extensors and hip flexors would result in significant muscle thickness and strength gains, and (2) these adaptations would correlate with enhanced balance performance.

## Methods

The study was designed as a longitudinal randomized controlled trial and data collection was performed over a period of three months. To investigate whether high-volume, high-intensity stretching can stimulate muscle thickness and maximal strength increases with transferability to balance performance, 49 participants were recruited and quadriceps thickness, dynamic balance and maximal isometric knee extensor strength were tested in a pre-posttest design. Participants were allocated via lottery to either the intervention or control group. Those participating in the intervention group completed twelve supervised 30-minutes stretching sessions using the ‘couch stretch’ (6 bouts of 5 min alternating legs). Neither participants, investigators, outcome assessors, nor data analysts were blinded to group allocation; except for the investigator who evaluated the ultrasound images.

### Participants

The minimally required sample size was estimated via G-Power 3.1.9.7 (Heinrich Heine University Düsseldorf, Germany) and returned *n* = 32 participants using maximal strength as the primary study outcome^28^ with d = 0.66 (f = 0.33). This calculation is based on assumptions of an α-error of 0.05 and a Power (1-β) of 95% for two groups and two measurement time points. To account for potential drop-outs and to enhance statistical power, an overall sample of 51 healthy and recreationally active participants (men: *n* = 30, age 23.7 ± 4.3 years, height 181.8 ± 8.8 cm, body mass 82.9 ± 11.1 kg; women: *n* = 19, age 23.4 ± 3.0 years, height 163.7 ± 6.1 cm, body mass 61.7 ± 8.2 kg) was recruited from the local university campus and sports clubs (Germany). Following randomization, the intervention group (IG) had a mean age of 24.4 ± 4.2 years, body mass of 74.1 ± 16.2 kg, and height of 173.6 ± 12.4 cm, while the control group (CG) had a mean age of 22.6 ± 3.1 years, body mass of 72.3 ± 10.8 kg, and height of 176.4 ± 10.8 cm. They were classified as recreationally active if they participated in sports university classes, worked out in a gym at least twice per week, stated that they regularly visit structured/organized sports programs (> 2x/week). If participants had previously performed (resistance) exercises, they were instructed to continue. However, they were not allowed to start or end their training and physical activity behavior throughout the intervention period. Thus, their exercise and physical activity routines had to remain the same as before the beginning of the study. Participants were excluded if they suffered a lower extremity injury within the 6 months prior to partaking in the study with these injuries entailing phases of immobilization, activity reduction, or other indications that could hinder a safe participation in the study. All participants provided written informed consent. The study protocol was performed in adherence to the declaration of Helsinki and was ethically approved by the institutional ethics review board of the Friedrich Schiller University Jena (No. FSV 24/104). The full trial protocol and statistical analysis plan are available at the German Clinical Trials Register (DRKS) (identifier: DRKS00036510). No changes were made to the protocol after trial commencement.

**Fig. 1:**
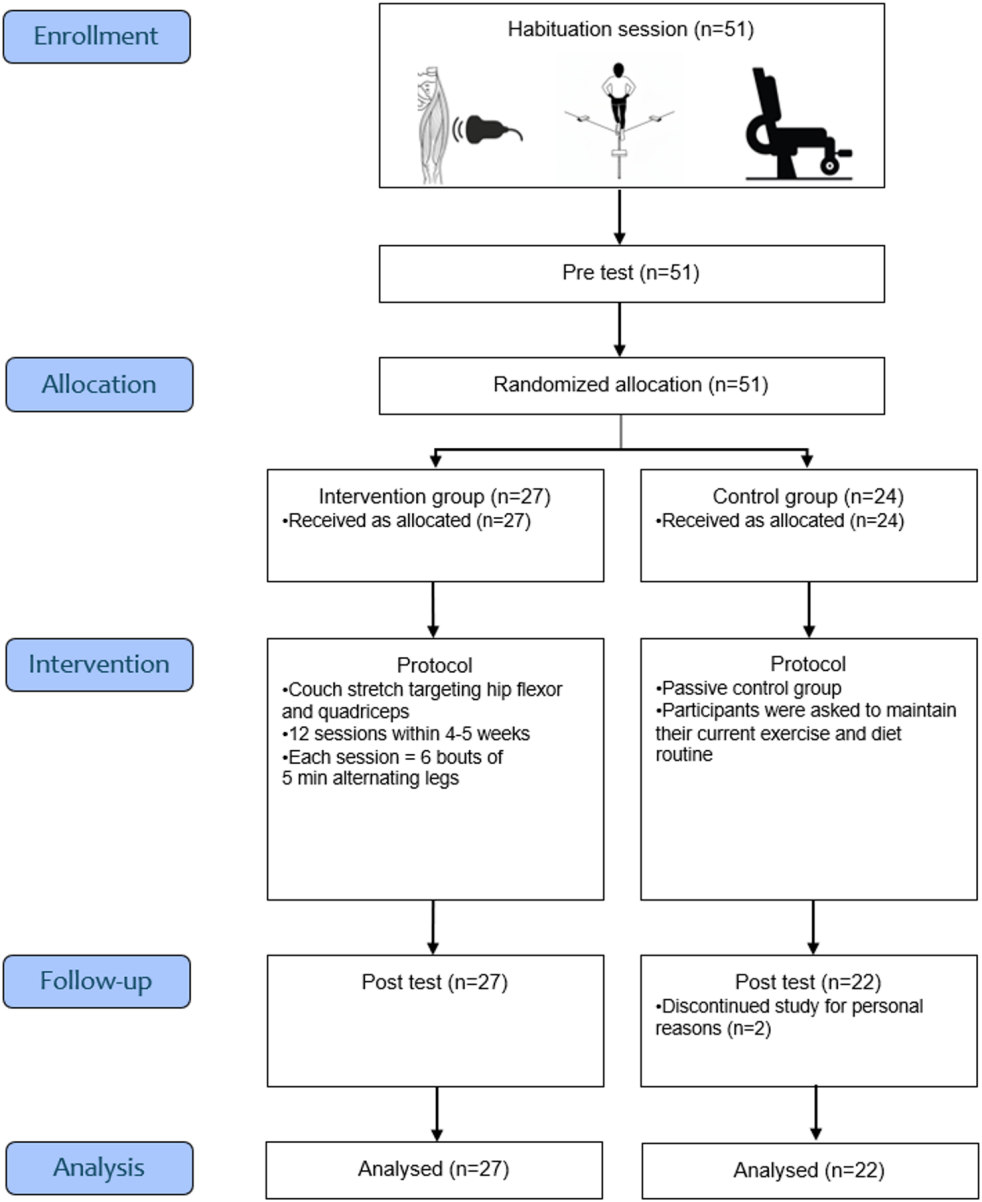
Flow diagram of participant enrollment, allocation, intervention, follow-up, and analysis*.*

### Testing protocol

The study consisted of three test sessions: a habituation session, a pre-test, and a post-test (see Fig. 1). Between pre- and post-test sessions, participants of the intervention group underwent a supervised stretching intervention. Before the pre-test session, participants visited the lab to address open questions, receive a detailed explanation of the study, and undergo a habituation session for the balance and isometric strength tests to minimize the influence of unfamiliar testing conditions^43^. Chronologically, muscle thickness, balance performance and isometric maximal strength were measured within all sessions. Randomization of the testing protocol was not performed as it was assumed that maximal strength testing could affect balance performance (e.g., due to fatigue) and each test should be performed under valid testing conditions.

### Ultrasound muscle thickness determination

Muscle size of the rectus femoris and the vastus lateralis was evaluated using B-mode ultrasound (Lumify, Software version 5.0, Philips Ultrasound LLC, Washington, USA). To account for potential regional differences in muscle size changes^44^, the proximal and distal portions of the rectus femoris were investigated. Standardization was performed by determining the transition from the muscle to the patella and taking muscle thickness pictures 5 cm proximal from this position. The more proximal part of the rectus femoris was determined by detecting the thickest position of the muscle belly via ultrasound imaging and marking this position with a water-resistant permanent marker. For the vastus lateralis muscle thickness determination, the middle between the distal and proximal rectus femoris spots was marked, going laterally from this position at a 90° angle from the line between distal and proximal rectus femoris spots. Standardization was ensured by measuring the distance of the distal and proximal measurement spots of the rectus femoris starting from the patella and noting these values for each participant. This served as a safety net to ensure standardization in cases where participants failed to repaint the ultrasound measurement spots during the invention period. Measurements were taken with a 5-cm linear probe and a measurement frequency of up to 24 Hz (see Figure S9 in the supplemental material). Imaging was conducted by a highly experienced investigator (> 12.000 ultrasound images in muscle morphology research settings)^45^. Ultrasound is the most common and most frequently applied method in literature to determine muscle hypertrophy, with most studies reporting sufficient reliability and validity^46^. Muscle thickness values were synthesized by an independent investigator who was blinded to group allocation of the participants. For muscle thickness, the mean of two images evaluated was used for further data processing^45^.

After finalizing ultrasound muscle thickness measurements, the participants were instructed to warm-up by walking for 5 min. Additionally, before performing the balance and the isometric testing, all participants performed a specific warm-up until they felt ready to reach maximal performance in the respective tests.

### Isometric maximal strength testing

The ISOMED2000 (D&R Ferstl GmbH, Hemau, Germany) was used to determine isometric strength in the knee extensor muscles at two angles to account for muscle length and joint angle specificity in isometric tests^43^. Since previous studies found strength increases only in high muscle lengths^32^, quadriceps strength was tested in 110° (lengthened position) and 70° (shortened position) of knee flexion. Each participant performed two sets of 3-second maximal voluntary isometric contractions (MVICs) on each leg. The device was individually adjusted to the participant’s anthropometry. The dynamometer’s axis of rotation was aligned with the lateral epicondyle of the femur. The padded lever arm was positioned so that its lower edge aligned with the level of the malleoli, providing resistance for the participants’ lower legs. To prevent upper body movement during testing, participants were securely stabilized using shoulder pads and a harness system. Participants were given time to warm up and familiarize themselves with the setup to ensure maximal effort during testing. For each trial, participants were instructed to push as hard and as fast as possible against the padded lever arm until the device signaled the end of the contraction. Verbal encouragement was provided by the investigator to motivate participants^47^. As we were interested in maximal strength values, the maximum of the test results was used for further data processing.

### Balance testing

The Y-Balance test (YBT) (Perform Better, Germany) was used to assess dynamic balance and stability. Participants stood barefoot on the central platform of the test kit and performed the test for both legs, alternating the sides to avoid fatigue. Each participant completed one practice trial per leg, followed by two measured trials. During the test, participants placed their hands on their hips and maintained their standing foot flat on the platform. They extended the other leg in one of three directions (anterior, posteromedial, posterolateral) to reach as far as possible without losing balance. At the end of each reach, participants were required to stabilize briefly in a controlled position. A trial was deemed invalid and repeated if any of the following occurred: hand(s) off the hips, heel of the standing foot lifts off the platform or foot of the contralateral leg touches the ground^48^. In valid trials, the distance was read off on the side of the apparatuses that face the participants. As we were interested in the best balance performance values, the maximum of the test results was used for further data processing.

### Stretching protocol

Stretching was performed for a total duration of 30 min per session, three times per week, resulting in 12 sessions over 4–5 weeks^40^. An intersession rest of maximal 72 h was allowed. The couch stretch was chosen for its anatomical specificity (hip extension with knee flexion) and ease of standardization in the laboratory. Participants were instructed to stretch both sides, each for 15 min which were split into 3 × 5 min. As Panidi et al.^41^ highlighted the necessity of high stretching intensities, the participants were instructed to perform stretching with a 8 out of 10 on the visual analog scale (VAS) with 10 indicating maximal tolerable stretching pain. If participants did not reach a sufficient stretching stimulus, foam pads were used to increase the stretching stimulus. Stretching was performed fully supervised in university premise. If participants reported a decrease in perceived stretching pain over the stretching bout duration, the supervisor of the stretching session increased the stretch stimulus by adding foam pads, as well.

### Statistical analysis

Data evaluation was performed using JASP (Version 0.18.3 (intel), Netherlands, 2024). Normal distribution was tested using the Shapiro-Wilk test. Descriptive statistics were provided as Means (M) and Standard Deviation (SD). Inter- and intra-day reliability was evaluated using the two-way intraclass correlation coefficient (ICC) for agreement between the habituation session and the pre-test, and the test-retest trial within the pre-test session, respectively. From ICCs, the Standard error of Measurement (SEM)^49^ and the Minimal Detectable Change (MDC)^50,51^ were calculated. To assess the relationship between maximum isometric strength and balance performance via YBT^21^, Pearson correlation coefficients (r) were calculated for the pre-test session performance between maximum isometric strength at short (70°) and long (110°) position of the stance leg in the YBT as well as for the reach distances in the anterior, posterior-medial, and posterior-lateral directions. Additionally, to test whether strength gains were associated with changes in YBT performance, we used repeated-measures correlation (rmcorr) to estimate the within-participant association between strength and balance across time points of the intervention group while accounting for between-subject variability^52^. A two-way analysis of variance (ANOVA) (Time $\times$ Group) with repeated measures was employed to address the main research question. Effect sizes were classified as follows: η_p_² <0.06, moderate: η_p_² = 0.06 - <0.14, large: η_p_² ≥0.14. The α-level was set to 0.05.

## Results

Two participants in the control group did not complete the study for personal reasons that were not further elaborated. No adverse events were reported during the study period. Normal distribution of data was ensured with *p* = 0.08–0.97 for all pre-test values apart from the YBT posterolateral reach while standing on the left leg which showed a p-value of 0.04.

Reliability values are provided in Table 1, reporting the intra-session reliability on the pre-test day as well as the inter-session reliability from the habituation session to the pre-test and account for repeatability as the precondition for internal data validity^53,54^. Relative reliability was classified as excellent for muscle thickness and maximal strength determination with the lower boundary of the ICCs 95% confidence intervals (CI) ≥0.9, while the YBT showed good to excellent reliability with the lower boundary of the 95% CIs ≥0.83^55^. For muscle thickness, SEM ranged from 0.005 to 0.015 with MDC of 0.013–0.050 across sides and regions. For maximum strength, SEM ranged from 0.718 to 1.468 (intra-session) and 1.339–2.463 (inter-session) with MDC values of 1.990–4.070 and 3.711–6.827, respectively. For the YBT, SEM ranged from 0.244 to 0.653 (intra-session) and 0.293–0.533 (inter-session), with corresponding MDC values of 0.676–1.809 and 0.813–1.476. Accordingly, changes equal to or greater than the reported MDC thresholds should be interpreted as true changes beyond measurement error.

**Table 1:** Showing the intra-session (during pre-test) and inter-session (between habituation session and pre-test) reliability using intraclass correlation coefficients with the respective 95% confidence intervals, standard error of measurement and minimal detectable change.

Parameter	ICC (95% CI) (intrasession)	SEM	MDC	ICC (95% CI) (intersession)	SEM	MDC
RF_Distal_R	0.96 (0.93–0.97)	0.014	0.050	0.96 (0.92–0.98)	0.014	0.039
RF_Proximal_R	0.96 (0.94–0.97)	0.011	0.031	0.98 (0.96–0.99)	0.008	0.023
VL_R	0.99 (0.99–1)	0.007	0.019	0.96 (0.92–0.98)	0.015	0.042
RF_Distal_L	0.98 (0.98–0.99)	0.005	0.016	0.96 (0.93–0.98)	0.011	0.030
RF_Proximal_L	0.99 (0.98–0.99)	0.005	0.021	0.98 (0.96–0.99)	0.007	0.019
VL_L	0.99 (0.98–0.99)	0.005	0.013	0.97 (0.95–0.98)	0.012	0.034
KneeExt_ short_R	0.98 (0.96–0.99)	0.718	1.990	0.97 (0.94–0.98)	1.357	3.762
KneeExt_ long_R	0.98 (0.97–0.99)	1.371	3.801	0.97 (0.94–0.98)	2.463	6.827
KneeExt_short_L	0.98 (0.96–0.99)	0.967	2.680	0.96 (0.93–0.98)	1.339	3.711
KneeExt_ long_L	0.97 (0.94–0.98)	1.468	4.070	0.97 (0.94–0.98)	2.194	6.080
YBT_Anterior_R	0.95 (0.91–0.97)	0.259	0.718	0.92 (0.86–0.95)	0.293	0.813
YBT_Posterolateral_R	0.95 (0.92–0.97)	0.514	1.425	0.91 (0.85–0.95)	0.525	1.455
YBT_Posteromedial_R	0.94 (0.90–0.97)	0.453	1.255	0.92 (0.87–0.96)	0.463	1.284
YBT_Anterior_L	0.96 (0.93–0.98)	0.244	0.676	0.90 (0.83–0.94)	0.328	0.909
YBT_Posterolateral_L	0.94 (0.89–0.97)	0.552	1.532	0.94 (0.90–0.97)	0.396	1.097
YBT_Posteromedial_L	0.93 (0.88–0.96)	0.653	1.809	0.91 (0.85–0.95)	0.533	1.476

Long = 110° knee angle, short = 70° knee angle, Anterior = anterior direction, KneeExt = knee extension, L = left leg, R = right leg, RF = rectus femoris, Posterolateral = posterolateral direction, Posteromedial = Posteromedial direction, VL = vastus lateralis, YBT = Y-balance test.

### Intervention effects evaluation

#### Maximal isometric strength in the knee extension

The four-week stretching program showed knee angle-specific strength increases. While in the right leg, there were significant time effects in both measured knee joint angles (η_p_²=0.13 and η_p_²=0.14, *p* = 0.013 and *p* = 0.01, respectively), no significant interaction effects were reported for the testing at 110° knee joint angle (*p* = 0.17 and *p* = 0.68). In contrast, moderate to large magnitude interaction effects (η_p_²=0.09 and η_p_²=0.21) indicated significant isometric maximal strength increases in 70° knee joint angle in the stretching group from pre- to post-testing in both legs (*p* = 0.04 and *p* = 0.001, respectively) (see Table 2 and Figure S5 and S7 in the supplemental material). Only the intervention group showed changes exceeding the MDC for isometric knee extension at the short position (70°) in both legs; in the control group, only the left leg at the short position decreased beyond the MDC.

**Table 2:** Showing the descriptives of the pre- and posttests for maximal isometric strength determination with the results of the 2-way analysis of variance (main and interaction effects).

Parameter	Pre-test (M±SD)	Post-test (M±SD)	Main effect	Time-Group Interaction
MVIC_long_R_IG [Nm] MVIC_long_R_CG [Nm]	159.96±51.88 158.26±52.48	166.64±54.98 163.16±51.99	F = 7.25 *p* = 0.01 η_p_²=0.14	F = 0.17 *p* = 0.68 η_p_²=0.004
MVIC_long_L_IG [Nm] MVIC_long_L_CG [Nm]	158.04±49.82 158.21±48.96	163.21±53.85 157.63±49.82	F = 1.27 *p* = 0.27 η_p_²=0.03	F = 1.99 *p* = 0.17 η_p_²=0.04
MVIC_short_R_IG [Nm] MVIC_short_R_CG [Nm]	241.18±82.16 232.58±70.44	259.29±80.84 234.47±69.76	F = 6.62 *p* = 0.013 η_p_²=0.13	F = 4.35 *p* = 0.04 η_p_²=0.09
MVIC_short_L_IG [Nm] MVIC_short_L_CG [Nm]	243.04±82.30 238.58±70.30	258.29±69.71 232.63±85.18	F = 2.27 *p* = 0.14 η_p_²=0.05	F = 11.81 *p* = 0.001 η_p_²=0.21

Long = 110° knee angle, short = 70° knee angle, CG = control group, IG = intervention group, MVIC = maximal voluntary isometric contraction, L = left leg, R = right leg.

### Quadriceps muscle thickness

Stretching increased the muscle thickness in both measured parts of the rectus femoris (proximal and distal muscle portions) with large magnitude interaction effects (η_p_²=0.14–0.28, *p* < 0.001–0.02). In contrast, in the vastus lateralis, effects were not as clear as for the rectus femoris: in the right leg effects did not reach the level of significance in the main as well as the interaction effects (*p* = 0.86 and *p* = 0.22). In contrast, in the left leg there were significant main (*p* = 0.011, η_p_²=0.16) and interaction effects (*p* = 0.008, η_p_²=0.18) (Table 3). All muscle thickness increases in the intervention group (rectus femoris distal/proximal and vastus lateralis, both legs) exceeded the MDC. In the control group, only the proximal rectus femoris (both legs) surpassed the MDC.

**Table 3:** Showing the descriptives of the pre- and posttests for muscle thickness determination with the results of the 2-way analysis of variance (main and interaction effects).

Parameter	Pre-test (M±SD)	Post-test (M±SD)	Main effect	Time-Group interaction
RFd _R_IG [cm] RFd _R_CG [cm]	1.78±0.44 1.83±0.49	1.99±0.39 1.82±0.45	F = 12.88 *p* < 0.001 η_p_²=0.26	F = 14.67 *p* < 0.001 η_p_²=0.28
RFd _L_IG [cm] RFd_L_CG [cm]	1.70±0.27 1.66±0.27	1.85±0.36 1.68±0.28	F = 24.21 *p* < 0.001 η_p_²=0.40	F = 12.02 *p* = 0.001 η_p_²=0.25
RFp_R_IG [cm] RFp_R_CG [cm]	2.37±0.43 2.50±0.51	2.61±0.49 2.54±0.49	F = 25.15 *p* < 0.001 η_p_²=0.41	F = 13.45 *p* < 0.001 η_p_²=0.27
RFp_L_IG [cm] RFp_L_CG [cm]	2.52±0.53 2.46±0.51	2.67±0.50 2.49±0.51	F = 11.34 *p* = 0.002 η_p_²=0.24	F = 5.95 *p* = 0.02 η_p_²=0.14
VL_R_IG [cm] VL_R_CG [cm]	2.81±0.58 2.75±0.58	2.86±0.58 2.71±0.58	F = 0.033 *p* = 0.86 η_p_²=0.04	F = 1.56 *p* = 0.22 η_p_²=0.04
VL_L_IG [cm] VL_L_CG [cm]	2.80±0.50 2.77±0.58	2.89±0.64 2.80±0.53	F = 7.08 *p* = 0.011 η_p_²=0.16	F = 8.00 *p* = 0.008 η_p_²=0.18

CG = control group, IG = intervention group, L = left leg, R = right leg, RFd = rectus femoris distal, RFp = rectus femoris proximal, VL = vastus lateralis.

### Y-balance test performance parameters

All but one (posterolateral right leg *p* = 0.07) measurements showed significant moderate to large magnitude increases from pre-to post (Time effect: *p* < 0.001–0.04, η_p_²=0.09–0.35) indicating improvements in both groups. Out of the six measures (three on the left leg, three on the right leg), there were three showing significant interaction effects (anterior and posterolateral left and posteromedial right leg) with *p* = 0.02 to 0.009 and moderate to large magnitude effect sizes η_p_²=0.13 to 0.14. The remaining tests remained without significant group-related pre-to post-test changes (*p* = 0.15–0.99) (see Table 4 and Figure S6 and S8 in the supplemental material). For Y-Balance performance, changes exceeded the MDC in the intervention group for the anterior and posteromedial directions bilaterally, and for the posterolateral direction on the left only. In the control group, only the right anterior and right posteromedial directions surpassed the MDC.

**Table 4:** Showing the descriptives of the pre- and posttests for balance performance measured via the Y-Balance test with the results of the 2-way analysis of variance (main and interaction effects).

Parameter	Pre-test (M±SD)	Post-test (M±SD)	Main effect	Time-group interaction
Ant_R_IG [cm] Ant_R_CG [cm]	56.39±6.57 57.59±6.67	58.43±7.66 58.84±7.24	F = 11.04 *p* = 0.002 η_p_²=0.20	F = 1.12 *p* = 0.30 η_p_²=0.02
Ant_L_IG [cm] Ant_L_CG [cm]	55.86±7.13 58.76±5.43	58.13±7.78 58.58±6.89	F = 4.63 *p* = 0.04 η_p_²=0.09	F = 6.42 *p* = 0.02 η_p_²=0.13
PostLat_R_IG [cm] PostLat_R_CG [cm]	99.54±12.56 100.18±10.99	100.91±11.29 101.58±11.72	F = 3.42 *p* = 0.07 η_p_²=0.07	F = 0.0017 *p* = 0.99 η_p_²=0.0
PostLat_L_IG [cm] PostLat_L_CG [cm]	98.46±11.29 100.74±9.21	101.61±9.57 100.95±9.57	F = 9.74 *p* = 0.003 η_p_²=0.18	F = 7.45 *p* = 0.009 η_p_²=0.14
PostMed_R_IG [cm] PostMed_R_CG [cm]	93.82±11.62 95.95±10.49	97.89±10.43 97.24±10.15	F = 23.86 *p* < 0.001 η_p_²=0.35	F = 6.43 *p* = 0.02 η_p_²=0.13
PostMed_L_IG [cm] PostMed_L_CG [cm]	95.64±11.12 96.32±11.64	99.21±10.60 97.47±11.38	F = 8.07 *p* = 0.007 η_p_²=0.15	F = 2.10 *p* = 0.15 η_p_²=0.05

Ant = anterior, PostLat = posterolateral, PostMed = posteromedial, CG = control group, IG = intervention group, L = left leg, R = right leg.

### Correlation between maximum strength and YBT performance and pre-post differences

Significant correlations were observed between isometric strength at short (70°) and long (110°) position and YBT performance (see Figure S1 and Figure S2 in the supplemental material). For posterolateral reach, isometric strength at long position correlated significantly with YBT performance on both the right (*r* = 0.467, *p* < 0.001) and left (*r* = 0.519, *p* < 0.001) side. Similarly, at short position, significant correlations were found for the right (*r* = 0.435, *p* = 0.002) and left (*r* = 0.465, *p* < 0.001) side. For posteromedial reach, isometric strength at long position showed significant correlations with YBT performance of both the right (*r* = 0.426, *p* = 0.003) and left (*r* = 0.424, *p* = 0.003) side. At short position, significant correlations were also found for the right (*r* = 0.379, *p* = 0.009) and left (*r* = 0.367, *p* = 0.011) side. For anterior direction, isometric strength at long position correlated significantly with YBT performance of the right (*r* = 0.374, *p* = 0.01) and left (*r* = 0.325, *p* = 0.026) sides. At short position, significant correlations were identified for the right (*r* = 0.401, *p* = 0.005) and left (*r* = 0.351, *p* = 0.016) sides.

Using repeated-measures correlation, pre-to-post changes in YBT performance showed positive associations with changes in isometric strength in a subset of pairings. On the left side, increases in short position strength were associated with greater anterior (r_rm = 0.446, *p* = 0.015) and posterolateral reach (r_rm = 0.384, *p* = 0.040). On the right side, increases in long position strength were associated with greater posteromedial reach (r_rm = 0.376, *p* = 0.044), with a non-significant trend for the short position (r_rm = 0.346, *p* = 0.066). All other strength-YBT pairings were not significant (see Table 5 and Figure S3 and S4 in the supplemental material).

**Table 5:** Repeated-measures correlations (r_rm) between maximal torque (long/short position) and Y-Balance Test reach distances (anterior, posterolateral, posteromedial).

Side	Variable A	Variable B	r_rm	*p*-value
L	YBT anterior max [cm]	Max force short position [Nm]	0.446	*p* = 0.015
L	YBT anterior max [cm]	Max force long position [Nm]	0.006	*p* = 0.976
L	YBT posterolateral max [cm]	Max force short position [Nm]	0.384	*p* = 0.040
L	YBT posterolateral max [cm]	Max force long position [Nm]	0.294	*p* = 0.122
L	YBT posteromedial max [cm]	Max force short position [Nm]	0.294	*p* = 0.121
L	YBT posteromedial max [cm]	Max force long position [Nm]	0.271	*p* = 0.156
R	YBT anterior max [cm]	Max force short position [Nm]	0.298	*p* = 0.117
R	YBT anterior max [cm]	Max force long position [Nm]	0.219	*p* = 0.254
R	YBT posterolateral max [cm]	Max force long position [Nm]	0.147	*p* = 0.448
R	YBT posterolateral max [cm]	Max force short position [Nm]	0.043	*p* = 0.824
R	YBT posteromedial max [cm]	Max force long position [Nm]	0.376	*p* = 0.044
R	YBT posteromedial max [cm]	Max force short position [Nm]	0.346	*p* = 0.066

## Discussion

This is the first study that showed significant stretch-mediated muscle thickness and strength increases in the quadriceps femoris. Furthermore, the observed correlation between YBT performance and quadriceps isometric strength highlights the potential role of muscle strength in enhancing balance ability. A recently published meta-analysis called for investigations regarding the possibility of implementing stretching in fall prevention programs in case of stretch-mediated strength increases would sufficiently moderate balance performance^36^. While some results point in a promising direction with some measures indicating significant improvements in balance performance, other results could not confirm these tendencies.

### Stretch-mediated hypertrophy and strength increases

Previous articles on stretch-mediated hypertrophy in the lower extremity focused on the plantar flexors^31,56^, while only one study performed quadriceps stretching over a period of six weeks and measured strength and hypertrophy within the last years^37^. We hypothesized that a lack of stretching intensity and volume could explain the missing significance in muscle hypertrophy in this study, as these moderators were highlighted in recent meta-analyses to be of crucial importance when inducing hypertrophy and strength increases in humans^40,41,57^. In accordance with the recommendations of Warneke et al.^40^ who suggested implementing a minimum stretching duration of 15 min per session with at least 3 sessions per week, this study adopted this training regime and increased the stretching duration in comparison with the Wohlann et al.^37^ study accordingly. While effects stay comparably small with muscle thickness increases of about 10–20 mm and strength increases of < 10%, the study design was sufficient to show that stretching can be used also in the quadriceps to increase strength capacity and muscle thickness.

Although RT is the most common form to increase strength and cause hypertrophy^15^, a demand for alternative exercise forms that can be implemented into daily routines was formulated in earlier research^29,58^. The most discussed contributor in RT to cause muscle strength and hypertrophy adaptations is mechanical overload^59^. This stimulus can be adjusted by adding weight to a bar or increasing the resistance of machines. However, also stretching can induce suprathreshold mechanical tension and cause similar physiological adaptations as observed in RT. Although underlying mechanisms were discussed in previous reviews, exact physiological mechanisms and pathways in human stretch-mediated hypertrophy remain speculative, as evidence majorly stems from animal research^60,61^.

Previous human studies focused on functional and morphological outcomes by using huge stretching volumes of up to 14 h per week^31^ for just one muscle group, limiting the practical applicability^34^. Therefore, the results of this study contribute to figuring out more economical stretching durations, finding a balance between invested time and resulting adaptations, as performing 15 min of stretching per leg, three times per week, over a duration of 4 weeks was sufficient to induce significant increases in muscle strength and hypertrophy.

However, the comparatively small effects regarding muscle hypertrophy and maximum isometric strength, especially in comparison to RT, can be attributed to several factors. The most obvious factor is the short intervention phase of only four weeks. Additionally, the intensity of the stretching protocol was quantified via subjective pain perception, making a progressive increase over the study period complicated. In such short training periods, it seems questionable if the effects can be specifically attributed to performing the stretch, or if it was just an unfamiliar stimulus. Future research is warranted in which stretch intensity is quantified objectively and the training protocol period should be extended to exclude effects which must be attributed to unfamiliar stimulation.

### Specificity in strength testing and regional hypertrophy

When testing strength there is a high task-test specificity. In this study we only tested for isometric strength. To increase internal validity^43^ and account for specific strength adaptation in different muscle length^32^, isometric strength was tested in the maximal muscle length installable in the isometric dynamometer (knee joint angle at 70°) and in a more shortened position (knee joint angle at 110°). The hypothesis was that stretching induces tension at high muscle lengths and would cause strength increases only at the 70° knee joint position, and not at the 110° position. This specific increase in strength production in high muscle lengths observed in previous studies^32,62–64^ could result from specific neuromuscular changes or be based on changes in the optimal muscle length for force production^62^. Indeed, RT studies found training in long muscle lengths induced strength increases when tested in high muscle lengths compared to short length partials^62,63^. The same was hypothesized for muscle hypertrophy, as Zabaleta-Korta et al.^44^and Nunes et al^65^. described that regional hypertrophy occurred and not all muscle parts respond to the same extent. Also here, numerous articles on performing RT in high muscle lengths induced regional hypertrophy in the distal part of the quadriceps^62–64,66^ and plantar flexors^67^. In contrast to the initial hypothesis and previous research^32,62–64^, there were specific strength increases obtained in this study, however, when tested in short muscle lengths, while no significant effects were found in the lengthened position testing.

These results and the underlying research methods do not allow to distinguish between specific neuromuscular effects caused by training in high muscle lengths, or if strength overall did not change with only the optimal muscle length for force production being altered. In this explanatory approach, it must be considered that most evidence refers to changes in the rate of force development rather than maximal strength changes^68–70^. Changes in the serial sarcomere number can be hypothesized when assuming a serial sarcomere increase in response to training in high muscle lengths as observed in animals^71^. Direct evidence in human studies is controversially discussed due to methodological limitations^60,72^. Instead, the fascicle length strength production relationship can be evaluated in future research^73,74^ and could help understanding stretch-mediated strength increases in high muscle lengths. Nevertheless, if stretching caused serial sarcomere accumulation, this would explain strength increases in high muscle lengths, but not in short. Further research including physiological and biomechanical evaluations will be necessary to explain the found results.

However, in line with the muscle thickness increase hypothesis, the vastus lateralis responded with a smaller and less systematic increase and underpins the relevance of regional muscle architecture/thickness evaluations^65^. In the rectus femoris, however, no significant differences were observed between the distal and proximal regions of the rectus femoris (both increased in size to a similar extent, apart from the proximal portion of the left rectus femoris).

As the quadriceps is a large, voluminous muscle group and it was speculated that the couch stretch majorly targets the rectus femoris in the more distal portions, ultrasound imaging was performed on two muscle locations in the rectus femoris as well as one position on the vastus lateralis.

### Balance performance

Balance performance is a multifaceted ability influenced by various factors, including lower limb strength^21^. While Turner et al.^75^ identified a significant variance explained by hamstring strength in YBT performance, our findings underpin the contribution of quadriceps strength to balance outcomes, emphasizing the importance of muscular strength in stabilizing the body during dynamic balance tasks. Our results stay in line with previous studies that examine the relationship between quadriceps muscle strength and balance ability in different tasks like the Biodex Balance System and functional reach test^76,77^. These findings form the basis for evaluating the impact of the stretching intervention on both strength and balance outcomes. As described, RT is a common intervention for increasing strength and muscle size and has been shown to be effective in improving balance performance^22^. This was acknowledged in current fall prevention guidelines^24^ recommending muscle strengthening activities as an intervention to improve balance ability in older adults. Although effective, RT has a limitation that hampers the practical application: Lacroix et al.^22^ found RT sufficient to moderate balance performance exclusively if it was supervised. As this could particularly affect restricted and/or immobilized populations with serious burdens, reduced commitment due to unsafe exercise conditions during unsupervised active training could hinder the effectiveness of common routines. If now stretching could provide an effective alternative for those with limited access to supervised RT routines or those who do not want to participate in structured RT programs, it could be a novel and easy applicable substitution in a home-based setting^35^. Unfortunately, the transferability of stretch-mediated strength increases remained uninvestigated, yet^36^. Therefore, with this study we sought to address this research gap by controlling both muscle strength, and balance performance. In our study, the YBT was used to map the balance ability of the participants. To achieve maximum YBT performance, participants must reach an optimal degree of knee flexion in the stance leg. Maintaining this knee flexion during the YBT relies heavily on the stabilization provided by the surrounding knee extensor muscles, as evidenced by the strong correlation between YBT performance and maximum isometric strength. Our intervention specifically targeted the knee extensor with the expectation of improving YBT performance. The results, however, open more questions than closing topics by pointing in promising directions. While in three out of six (anterior left, posterolateral left and posteromedial right leg) measures significant interaction effects indicate improved YBT performance after the intervention period, in all tests, the intervention group started on lower baseline values compared to the control group and both groups finished on almost the same level. Thus, the clinical relevance of these balance improvements remains uncertain. Additionally, the observed effects are relatively small, likely due to the short four-week intervention period. However, repeated-measures correlations revealed a few significant, small-to-moderate associations between pre-post changes in YBT performance and isometric strength, though the pattern was inconsistent across sides and positions. It is noteworthy that this period included an extensive high-volume stretching protocol, exceeding previous quadriceps stretching studies^37,78^. Furthermore, other limiting factors influencing YBT performance, not targeted or measured by our intervention, must be considered. Previous research has shown that YBT performance is affected by dorsiflexion, as well as knee and trunk flexion abilities^75,79,80^, which may explain the significant but modest effects observed in this study. Future studies should aim to investigate the variance explained in YBT performance by incorporating a broader range of strength and flexibility variables.

### Practical relevance

The present study represents proof-of-principle work designed to examine whether stretch-based loading can induce adaptations in the knee extensors. Although the intervention was tested in young, healthy individuals, such baseline research is a prerequisite before translation to clinical populations. The findings provide preliminary evidence that passive stretching can modulate muscle morphology and strength, supporting the concept that stretch-mediated mechanical stimuli can elicit muscle adaptations, whereas transfer to balance tasks remained uncertain. While direct applicability to rehabilitation or fall prevention cannot be inferred from the present data, these results establish a foundation for future studies in older adults and clinical populations. Further research is required to determine optimal dose-response relationships, identify the muscle groups most responsive to this intervention, and evaluate its feasibility and effectiveness in clinical settings.

### Limitations

As all balance tests, the YBT is considered a highly specific skill^81–83^. Therefore, the results cannot be generalized to other balance assessments. Additionally, attributing the observed (small) balance improvements to strength increases is challenging, as stretching might also caused ROM increases that were not controlled in this study. Another limitation of this study is the comparatively short intervention period, allowing not to distinguish between effects that could be attributed to unfamiliar stimuli and whether stretching provides a sufficient mechanical overload to induce chronic muscle hypertrophy and strength increases. The choice of intervention exercise presents another limitation, as one leg was stretched, the other leg had to keep the body weight upward and was therefore stimulated. However, as all participants stated to regularly exercise and were classified as active participants, it seems unlikely that stabilizing the bodyweight in a kneeling position could cause muscle thickness increases. Though, since RT experience was not specifically queried in this study, future research should further distinguish the training status upon enrollment. Finally, the variability in participants perceived stretch location, with some reporting it in the hip region and others closer to the area above the patella. Since this was not systematically assessed, future studies should investigate how stretch localization affects regional hypertrophy - and thus also investigate different portions of the vastus lateralis. Additionally, the assumption that the rectus femoris experiences the greatest tension during the stretch was not directly tested and warrants further exploration. A passive nature of the stretching stimulus was assumed, however, without direct evidence in humans. While animal studies suggested that muscle activation was not increased while stretching^84,85^ or required to cause muscle hypertrophy^86^, no such studies were found for humans and call for caution in interpretation.

## Conclusion

This study provides first evidence for stretch-mediated hypertrophy and strength increases in the quadriceps, while the influence of balance performance remains unclear. Nevertheless, the study underlines that, in contrast to previous articles, one hour of stretching is not necessary to increase strength and muscle size which improves the practical relevance of this intervention. Nevertheless, results are biased by a short intervention period, calling for future research in this area that apply stretching for different intervention periods, stretching durations and frequencies to provide further insights into this young research area.

## Supplementary Information

Below is the link to the electronic supplementary material.

Supplementary Material 1

Supplementary Material 2

## Data Availability

Original data can be requested from the corresponding author due to reasonable request.
