# Supplementary material for: Stretch-mediated hypertrophy and strength increases and their impact on dynamic balance performance - a randomized controlled intervention study
Source: Sci Rep. 2026 Mar 9;16:8482. doi: 10.1038/s41598-026-43038-1 (PMC12972296; doi:10.1038/s41598-026-43038-1)
Supplement: Supplementary file 1 — Supplementary Material 1 [file 41598_2026_43038_MOESM1_ESM.docx]

**Supplemental Material**

**
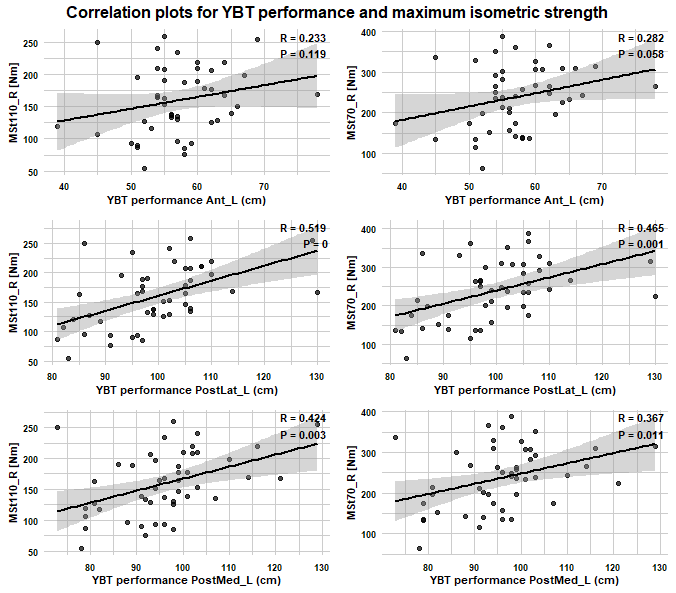
**

**Figure S1** *Correlation plots for the maximum isometric strength at 110° and 70° knee angle of the right side (stance leg in the YBT) and YBT performance in anterior, posteriorlateral and posteriormedial direction*


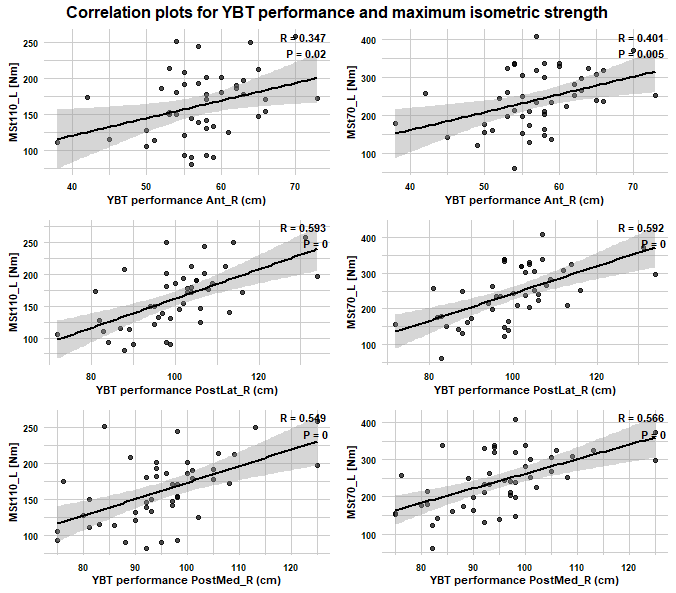


**Figure S2** *Correlation plots for the maximum isometric strength at 110° and 70° knee angle of the left side (stance leg in the YBT) and YBT performance in anterior, posteriorlateral and posteriormedial direction*


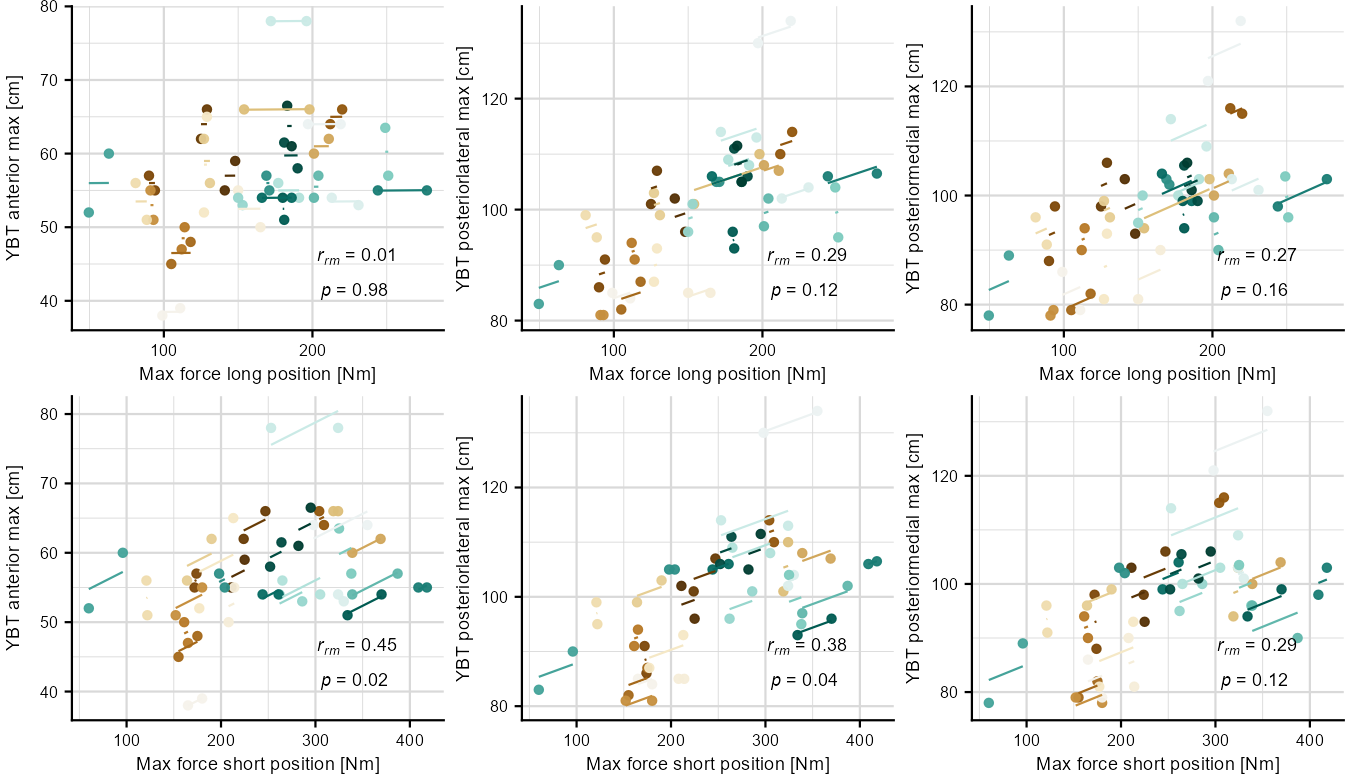


**Figure S3** *Repeated-measures correlations between maximum isometric strength and Y-Balance Test performance for the left leg.*


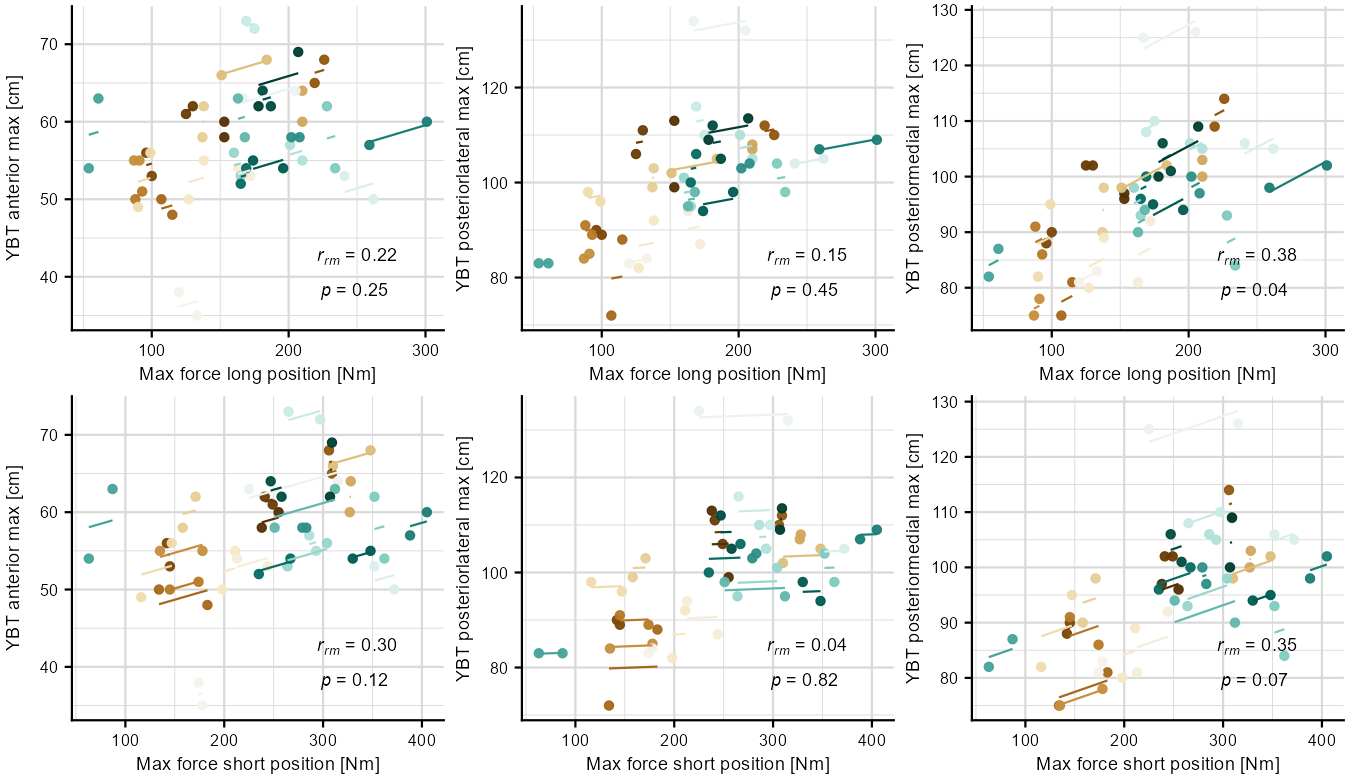


**Figure S4** *Repeated-measures correlations between maximum isometric strength and Y-Balance Test performance for the right leg.*


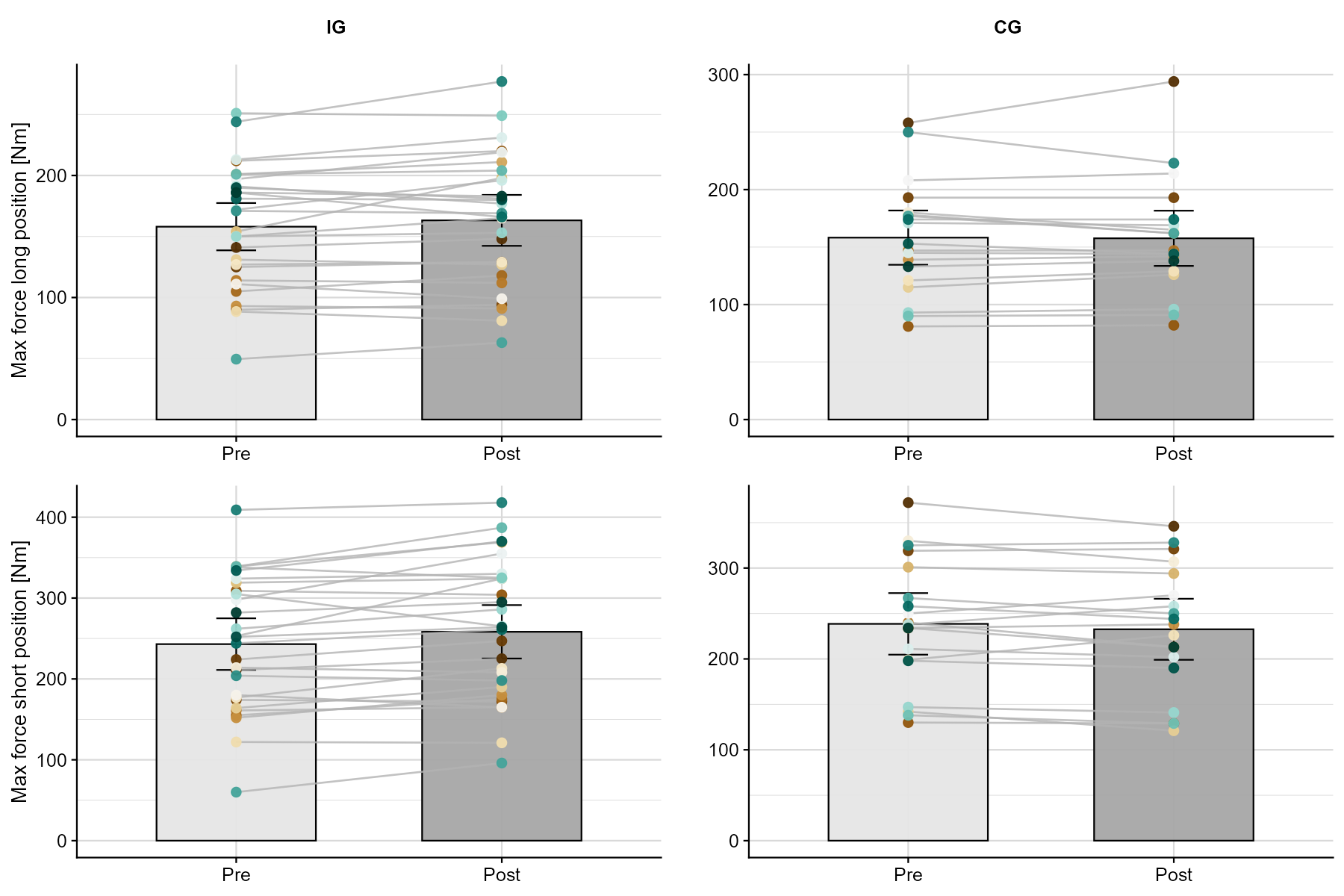
**Figure S5** *Pre-post changes in maximum isometric strength at long and short positions of the left leg*


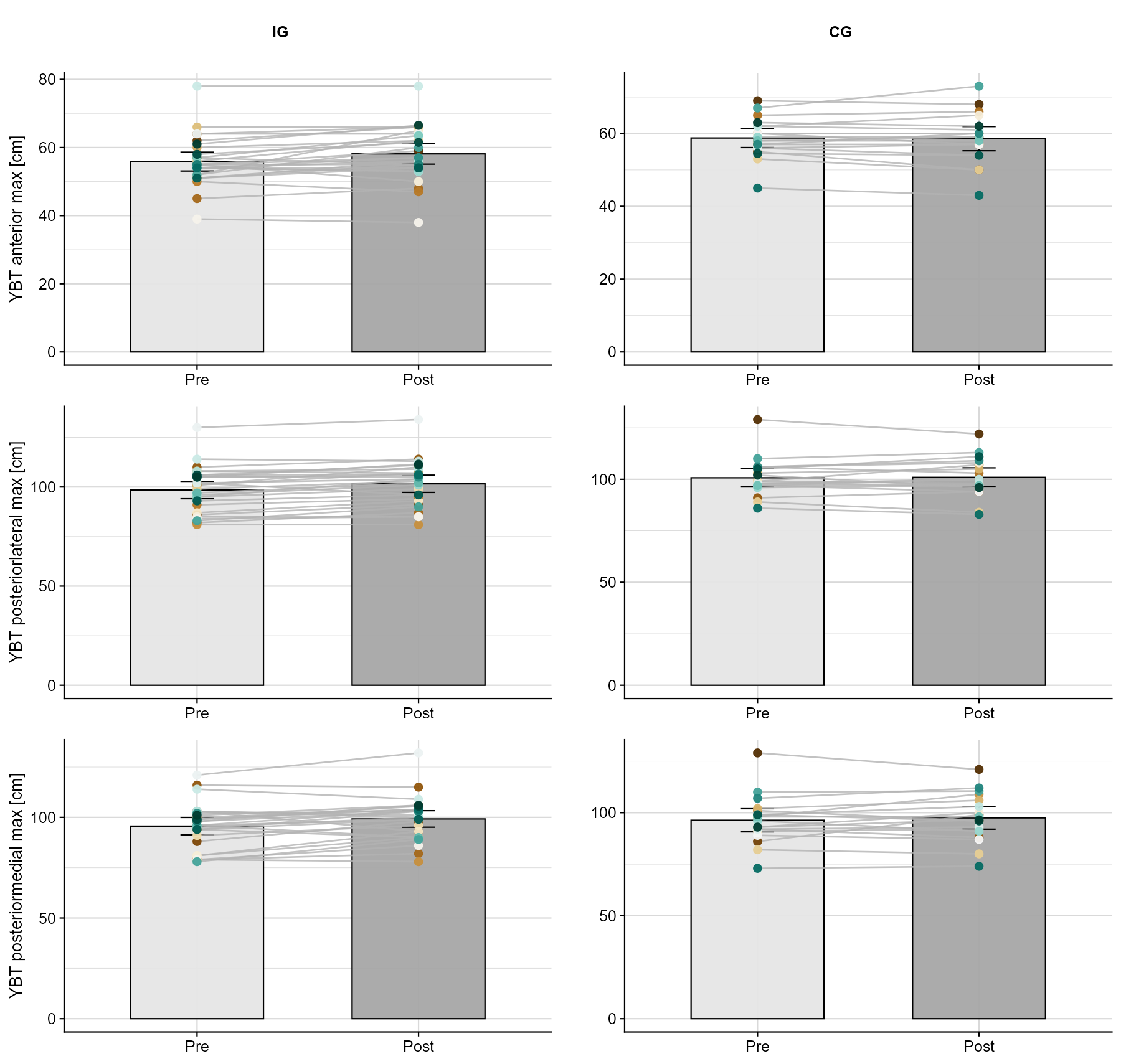
**Figure S6** *Pre-post changes in Y-Balance Test (YBT) performance of the left leg*


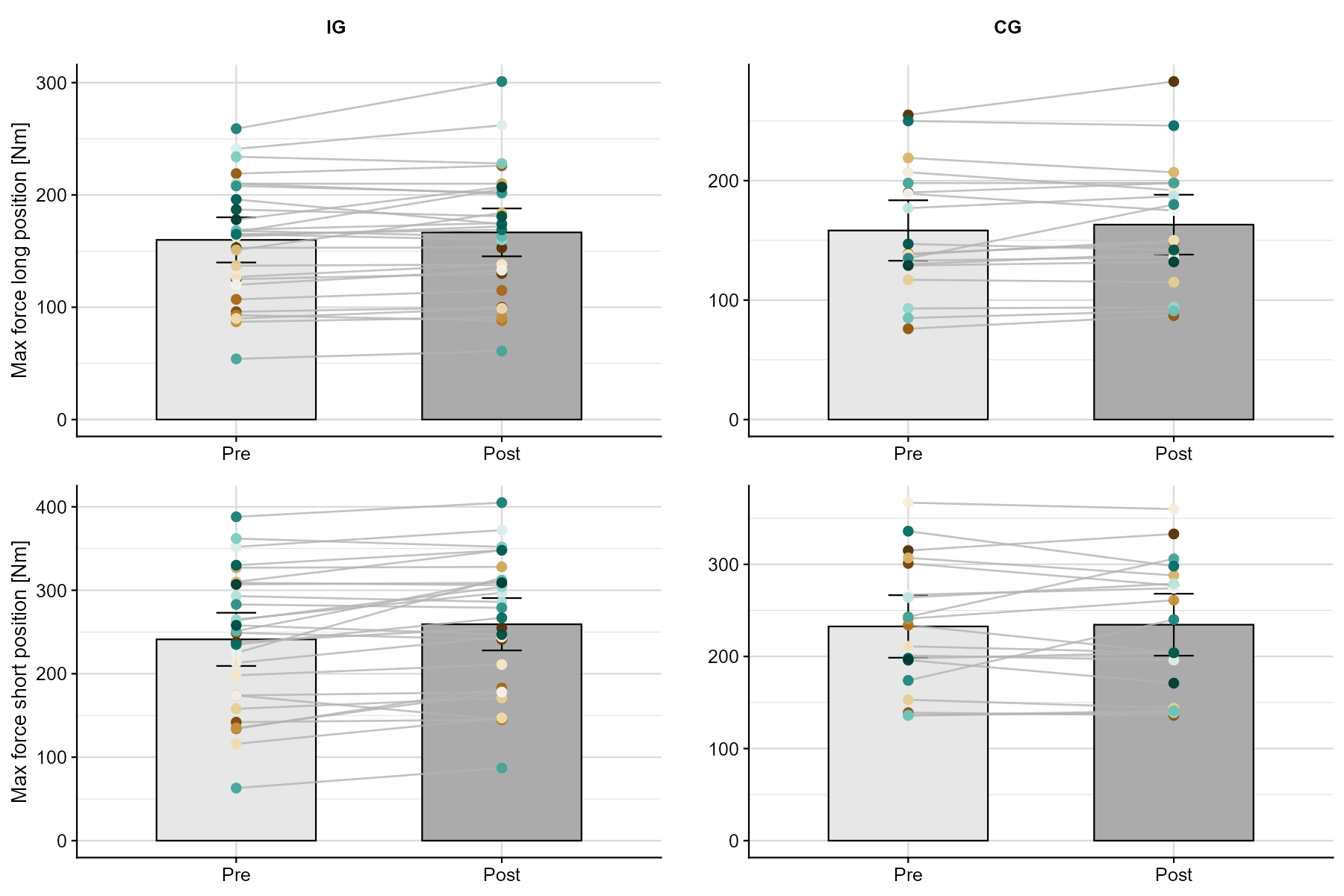
**Figure S7** *Pre-post changes in maximum isometric strength at long and short positions of the right leg*
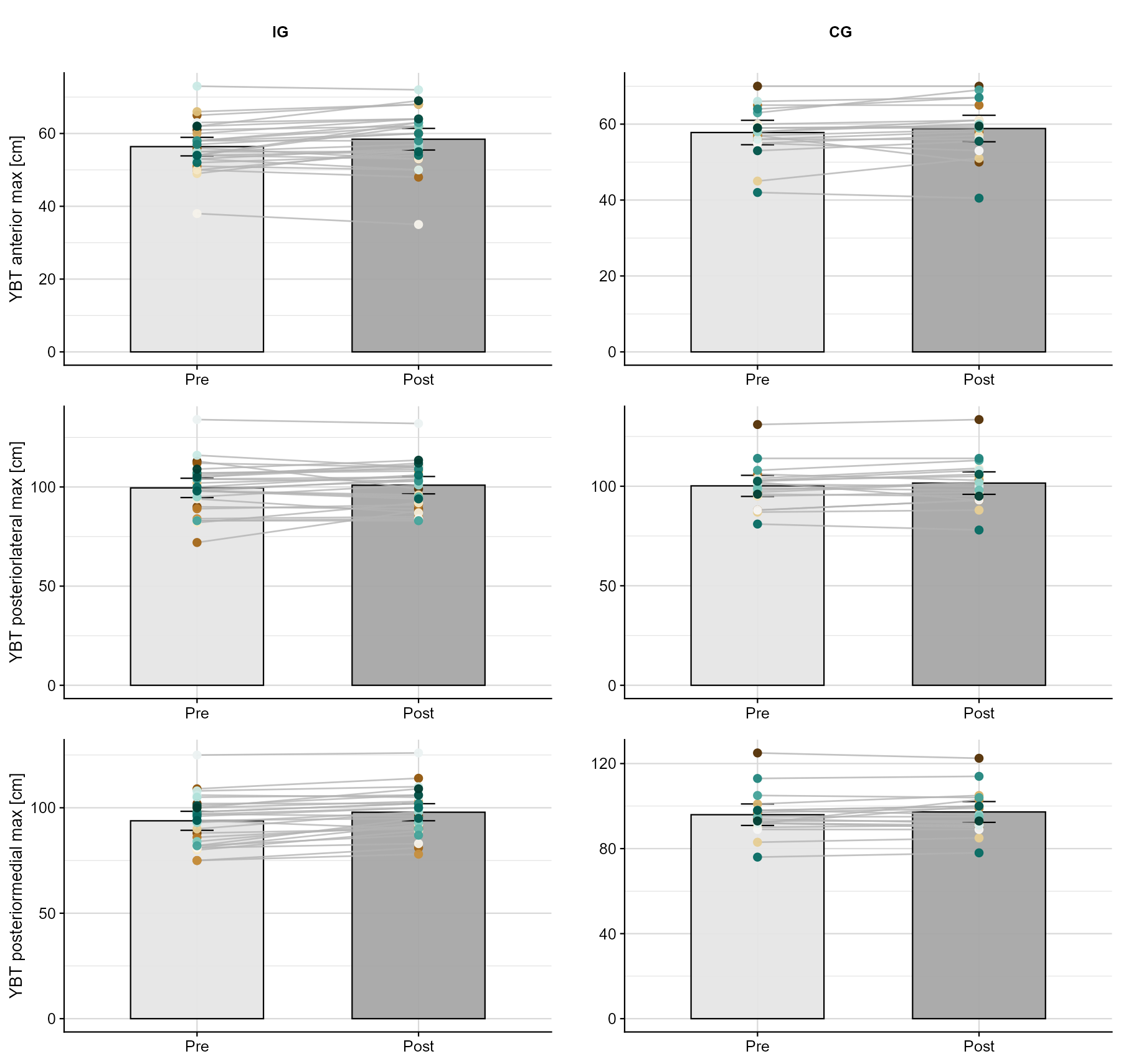
**Figure S8** *Pre-post changes in Y-Balance Test (YBT) performance of the right leg*


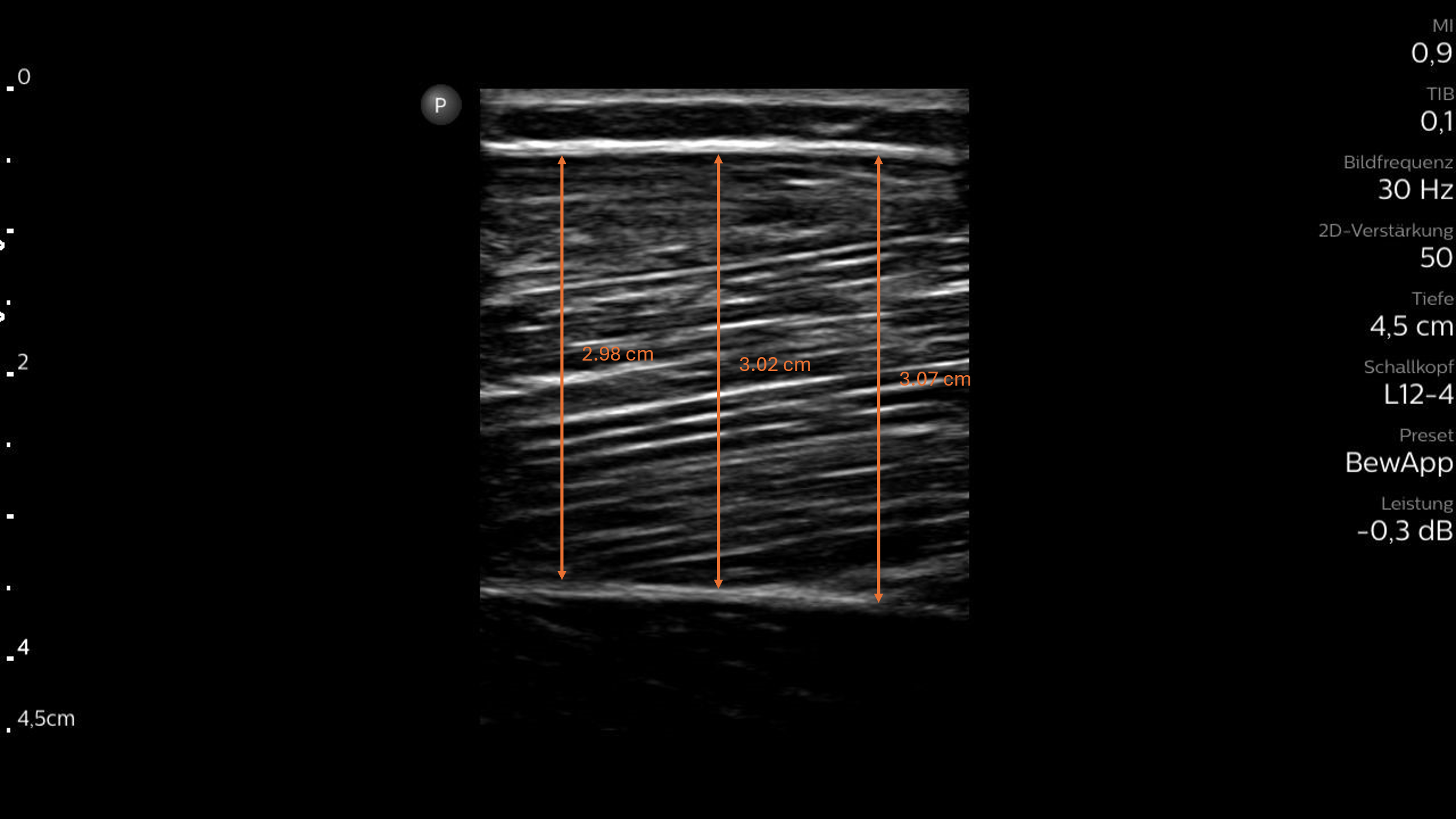


**Figure S9** *Example of an ultrasound image of the vastus lateralis*
